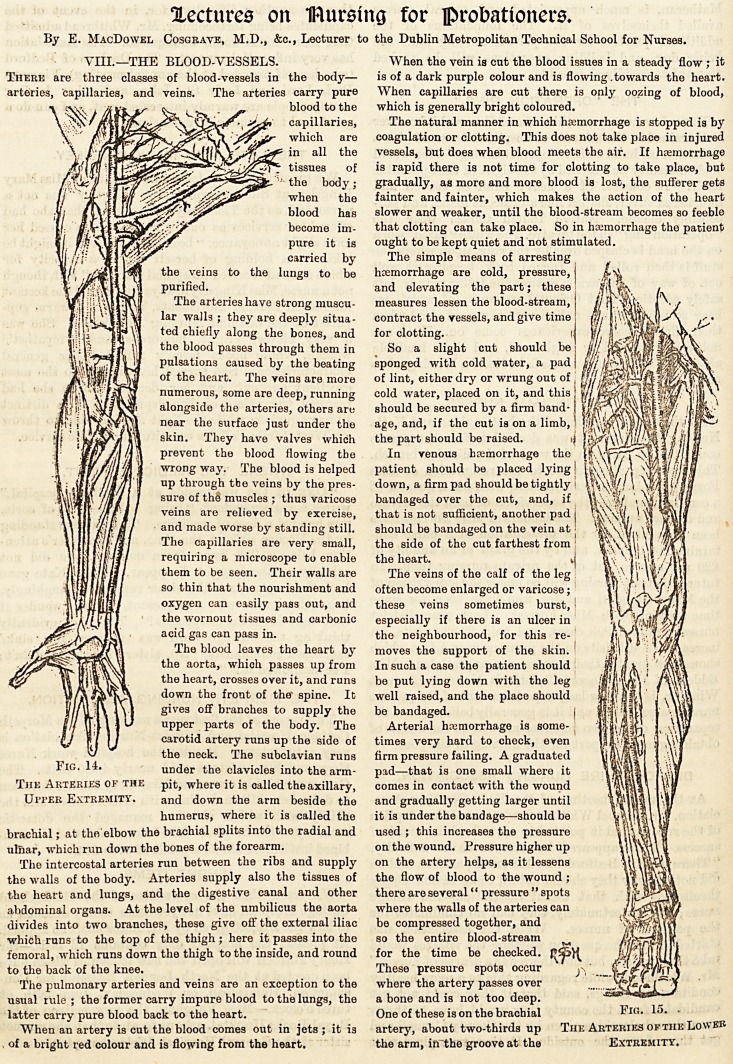# "The Hospital" Nursing Mirror

**Published:** 1900-06-09

**Authors:** 


"The Hospital" Nursing Mirror.

Being the Nursing Section of "The Hospital."

[Contributions for this Section of "The Hospital" should be addressed to the Editor, The Hospital, 28 & 29, Southampton Street, Strand, London, W.C., and should have the word "Nursing" plainly written in left-hand top corner of the envelope.]

### Notes on News from the Nursing World.

### IMPROVEMENTS AT ST. GEORGE'S INFIRMARY.

We learn with much satisfaction that the efforts in which we took some part, on behalf of the nurses of St. George's Infirmary, Fulham Road, are bearing fruit. A former member of the staff assures us that "a great many changes are about to take place." The night nurses are already having shorter hours of duty, and they are allowed to be off duty every third Sunday. The staff has been greatly increased, and day nurses are having more off duty. As to the patients, there is nothing definitely settled, but it is anticipated in the infirmary that the children will shortly be removed to separate wards, and that the diet of the patients will be improved. We sincerely trust that these prognostications will be speedily justified by events. The card which every lady visitor is requested to sign contains no unreasonable conditions; and, altogether, there seems ground for the belief that the Guardians of St. George's, Hanover Square, while protesting they would ne'er consent, consented—to reform.

### THE CORK GUARDIANS AND PROBATIONARY NURSES.

The Cork Board of Guardians have under consideration a scheme for the training of probationers in the workhouse, but some of the Guardians are so bitterly opposed to it that there is a doubt whether Cork will have the good sense to emulate the excellent example of Belfast. The conditions on which the Irish Local Government Board have recognised the Belfast Workhouse as a clinical hospital are as follows:—

"(*a*) Probationers to be appointed by the Guardians subject to the following: (*b*) Probationers must pass a physical examination by medical staff; (*c*) must satisfy medical staff as to a certain standard of educational ability; (*d*) the age limit to be from 20 to 27 years, and the period of training to be three years, divided as follows: Two years in general hospital, six months in fever hospital, and six months in the maternity. After three months' residence the medical staff are to report to the Board of Guardians as to the progress made, and as to the suitability of each probationer. In the event of the report being unfavourable at this period, or at any further period, the Board are to dispense with her services. The probationers are to be accommodated in a nurses' home, to be provided by the Guardians, and to be under the charge of a nun specially appointed, who will be responsible for their conduct and assist in their training. Terms: To first year probationer, no remuneration, except a suit of uniform and rations; second year, £8, with rations; third year, £14, with rations. Training of nurses: Lectures to be given by the medical staff, in addition to instruction in the wards by surgical and nursing staffs. Examinations to be held half-yearly by the medical staff of the workhouse."

The need of some drastic alterations in the Cork Infirmary is proved by the statement of Dr. Browne, the Local Government Board Inspector, that there are at least 70 pauper attendants nursing the sick! But there are local Solons who talk about the Guardians "putting their foot down," and not allowing the Local Government Board to force them to further expenditure. The Chairman is not one of these, and he favours the scheme for training on the ground that "it would afford young girls an opportunity of earning a respectable living at home, and keep them from going away in the emigrant ship."

### THE UNIFORM QUESTION AND THE BRITISH NURSES' ASSOCIATION.

At the annual meeting of the Royal British Nurses Association, which takes place on Saturday, June 23rd, it is proposed to discuss the question of protection for the uniform of nurses. Whether the suggestion which appears in another column, by a correspondent who is a member of the Association, will be acted upon remains to be seen. But the idea of trying to obtain an Act of Parliament for the purpose is not likely to obtain much support. Nurses have nothing whatever to gain from the intervention of Parliament; and if wearing the uniform by a person not belonging to the profession were made an indictable offence, the difficulties of detection would be insuperable. For a policeman to be able to stop anyone attired as a nurse whom he suspected of being an impostor, and ask for proof that she was not, would open the door to more unpleasant contingencies than at present exist.

### REFUSING A NURSE BANDAGES.

It appears that the master of Bridgend Workhouse has been guilty of a more serious offence than refusing to allow the nurses starch in their aprons. The *Western Mail* reports that at a meeting of the Bridgend Board of Guardians last week the House Committee, after holding a special meeting relative to a series of charges preferred by Nurse Wilson against the master and matron of the house, recommended that the master be severely reprimanded for his refusal to supply the nurses with bandages when asked for. The case was that on May 19th a man was admitted to the house suffering from broken ribs, and on the Thursday the nurse applied for bandages, but the master refused to give her any, and on Saturday evening the man died. "The chairman," says our contemporary, "in reprimanding the master, said he would not press the matter, but leave it to his own conscience." We are not sure that a reprimand was in this instance sufficient. An infirmary official who declines to supply a nurse with bandages when she requires them is the wrong man in the wrong place.

### THE EIGHT HOURS' SYSTEM IN NEW ZEALAND.

Although the Hospital and Charitable Aid Board of Auckland have postponed taking any steps in the matter of an eight hours' system until the Nurses' Home has been enlarged, and adequate accommodation provided for an increased staff, the discussion on the question at the last meeting was almost entirely favourable to the principle. In fact, absolute hostility to it was only expressed by one speaker. At present there are 45 probationers in the hospital, and it is estimated that under the new scheme 55 would be required, the extra annual outlay being approximately £1,000. This means that the ratepayer will have to find £500 and the Government £500. As Mrs. Grace Neill, the Deputy-Inspector of Lunatic Asylums in New Zealand, said last summer, when she was in London, the system has "many disadvantages," but her defence of it was that "in a new country like New Zealand it seemed the only course open." From the nurses' point of view, the reduction of twelve to eight hours, which is becoming general, is certainly not a disadvantage.

### THE NURSES OF PLYMOUTH INFIRMARY.

Last week we published the remarkable letter in which the nurses employed at Plymouth Workhouse Infirmary recited their reasons for dissatisfaction with the arrangements of the Guardians for their comfort or discomfort. In our opinion they have made out an unanswerable case for reform, and have shown in a clear and convincing manner why there have been so many resignations of the nursing staff. They have a right to ask for the provision of a sitting-room which is not a common dining-room; to expect that their meals shall be served punctually; to privacy, proper ventilation, and freedom from offensive smells in their dormitories; and to adequate bath and lavatory accommodation. It should also be easy to afford protection against draughts and rain in the yards between the blocks; and the outdoor nurses are certainly justified in complaining of the absence of a room for changing their wet clothes. It cannot be impossible to find them accommodation in the workhouse. Plymouth is such a prosperous and important town that we cannot believe its citizens will long submit to a state of affairs which would be unworthy of the veriest Little Pedlington.

### THE TRAINING OF MALE NURSES.

Every two years eight male nurses are trained at the National Hospital for the Paralysed and Epileptic, Queen Square, Bloomsbury. They have the entire charge of two wards, and they also perform certain duties with regard to all the male patients, of which massage is one. When the term of training has been satisfactorily completed, the male nurses find no difficulty in obtaining remunerative employment. In massage they are specially useful. Besides the regular course of instruction to nurses, there are daily massage classes for outside pupils, to whom certificates are granted when proficient. There are always plenty of applicants for training, whilst the daily massage classes are largely attended.

### THE NURSES' HOME AT LAMBETH INFIRMARY.

In the circumstances, it has been very properly decided by the Lambeth Board of Guardians to erect a nurses' home above the offices. Originally it was proposed to build one on the site of the old mortuary, but it was objected to by the Local Government Board on both administrative and architectural grounds. The choice then was limited to the removal of the offices to another site, and the erection of the nurses' home in their place, and the scheme which has been adopted on the ground that it involves far less expenditure. It is the duty of Guardians to observe economy whenever it is consistent with the best interests of the community; and there does not appear to be any objection to the plan selected on the ground of unsuitability.

### THE NURSES AT THE NEW ALEXANDRA HOSPITAL.

The beautiful new hospital built for the little patients suffering from hip disease has now been in occupation for some months. One large and one small ward are arranged on each of two floors, and give accommodation for 64 beds. Above are the nurses' bedrooms, large pleasant apartments, well furnished, and comfortable. Each has two single beds, two combination dressing-chests, and one large wardrobe with two compartments. The floors are covered with cork carpet, and the decorations are in excellent taste. One room has been fitted up for music and social recreation. It is sufficiently far away from the wards to allow of the nurses indulging in music and merriment without fear of disturbing their small charges. In the evenings one of the waiting-rooms is used by them for reading and rest. The children seem to enjoy life in spite of being tied upon their back for months, or even years, as the case may be. Nurse Amy is a special favourite, partly because once upon a time she, too, was a patient; and getting quite well and strong, came back to the hospital to nurse, just as she always longed to do. "And," the children say, "if Nurse Amy got well we will get well too, and if we can't all be nurses we shall be able to walk about like other people."

### A UNIQUE PATIENT.

"This afternoon," writes a nurse at one of the largest London hospitals, "about half-past four p.m. a Scotch terrier limped into the main entrance of the hospital, sat down on the mat holding up a wounded paw, and mutely appealed to a group of students standing in the hall. One of them lifted him up, brought him into the casualty ward, and laid him on a couch. At once a crowd gathered to have a peep at the four-footed patient, who lay down, quietly wagging a very stunted tail, holding up his paw, and looking as self-satisfied as any dog could who had hit the nail on the head and done the right thing. On examination a small discharging abscess was discovered, which was syringed with 1 in 80 carbolic acid lotion, and dressed with a boracic fomentation. All this time the dog was as immovable as the Great Sphinx. He let us stroke him down and pat his head, and after a little coaxing condescended to eat a biscuit piecemeal. His address was written on his collar, so one of the students offered to carry him home. We could not enter him in the book, but we did tie a casualty card to his collar, and told him to come to the out-patient entrance the next morning at nine a.m."

### THE NURSES OF ST. GEORGE'S HOSPITAL, BOMBAY.

A very interesting report has been issued by the sister in charge of St. George's Hospital, Bombay. The numbers at present are: Private nursing staff, 16; hospital service, 15: juniors, 6; probationers, 7. Last year the number of patients nursed were 211; of which 33 were midwifery and 6 plague cases. The results of the examination during the year were that 11 were sent up for the senior, of whom 1 only failed and left; 7 went up for the final and were all successful, and of these 5 are now working on the private nursing staff. The sister in charge considers this is largely due to Major Hojel, who has taken unfailing interest in the training of the nurses. The grant made by the committee for nurse's "leave," spent at Khandalla or

Matheran, is much appreciated, and several nurses availed themselves of it. The foundations of the additional quarters for the private nursing staff are being dug, and building operations will be pushed forward as rapidly as possible.

### THE "OPERATION RING."

Very few nurses on this side of the world, however well trained in the *minutiæ* of hospital life, could give a definition off-hand of the "operation ring." Nor would their difficulty be lessened by the sight of the apparatus itself. It consists of a heavy band of carved and hammered silver, shaped like a crescent moon, and perceptibly elastic. Native Indian nurses wear the "operation ring" in the theatre. The white "saree" worn on the head is clasped over by the ring, upon which the stuff is then rolled and turned back, keeping it well out of way of the arms, so that the latter may be left safely aseptic. That Oriental calmness of which we hear so much stands native nurses in good stead when their first theatre experiences come on. They are not easily flurried or frightened, and their laudable absence of self-consciousness is in itself a blessing of no small value.

### THE NOTTINGHAM NURSES.

At the annual meeting of the Nottingham and Notts Nursing Association it was decided to form a separate committee to carry on the work of the district branch. This new departure is due to the resignation of Miss Forrest, the lady superintendent, who, for a quarter of a century, combined the supervision of both the private and district nursing. The labours of Miss Forrest have been recognised by the presentation of a purse containing 200 guineas, and by the grant of an annuity of £20 per annum out of the private nursing fund. In future, district nursing will be under the supervision of the Misses Stendall and Jee, who have been for some time working with Miss Forrest. Last year district nurses attended 905 cases, and paid 39,889 visits, an increase of 8,404 visits on the previous year, a fact which shows how greatly their services are appreciated. Miss Edden, of the Princess Christian's Nursing Home at Windsor, is the new lady superintendent of the private nursing association, and it is generally believed that the separation of the two departments will be the means of obtaining more support for the nursing of the poor.

### BEDFORDSHIRE NURSING ASSOCIATION.

At the annual meeting of the Beds. Nursing Association, Mr. Samuel Whitbread, who moved the adoption of the report, said it pointed to an unbroken record of success. There appeared, however, to be one difficulty. "There were in Bedfordshire 128 rural parishes, and he did not see why they should not all come in and supply themselves with that useful institution—the village nurse; but, unfortunately, they had no candidates for the position of nurses. When the association was started it was the question whether the villages would take their nurses, but now there was a dearth of nurses." Mr. Whitbread, while regarding this as a more healthy condition of things, said that if they could not get candidates from the county they would have to throw open the scholarships given by the County Council and get them from the outside. It then transpired that there is another difficulty; for, in the event of the candidates being forthcoming, Mr. Whitbread admitted that more funds would be required. The Association has very influential support. The Duchess of Bedford (who herself presided at the annual meeting), Viscount Peel, Lord and Lady St. John, and other leading county people are warmly interested in it, and can do a great deal to promote its extension.

### THE LATE MISS MARY KINGSLEY.

We greatly regret to learn of the death of Miss Mary Kingsley at Simonstown. Miss Kingsley was not a nurse, and, as the *Times* says, the report that she had offered her services as one in South Africa caused her some slight annoyance, "because she feared it might be taken as a holding of herself out in a capacity for which she had received no special training." But, though not a nurse, Miss Kingsley always manifested the keenest interest in nursing movements, and was a warm supporter of the Colonial Nursing Association. She was a very clever, bright, humorous, and sympathetic woman, whose books largely reflected her general characteristics. She turned her travels to the most excellent account, and her decease before she had reached the prime of life deprives us of a distinct literary genius; while the light she was able to throw on West African affairs constituted a public service.

### WHERE THE MEDICINE WENT.

"One of the wardmaids at a Midland hospital," writes a correspondent, "being a little out of sorts, was handed a dose of medicine. The girl was standing by the kitchen sink at the time, and the sister's attention being called away for a moment, she did not actually see the draught disappear. When Kate gave back the empty glass, the sister remarked, laughingly, to another nurse who was present, 'Now, I wonder if that went down the œsophagus?' The maid, evidently thinking the strange word was 'foreign' for 'sink,' exclaimed indignantly, 'No, sister, indeed it didn't; I've swallowed it!'"

### THE MORPETH NURSING ASSOCIATION.

The report of the first year's working of the Morpeth Cottage Hospital and District Nursing Association is very satisfactory. Besides the hospital work Nurse Grey and her assistant paid nearly 4,500 visits. The Governors highly commend Miss Grey for her devotion to duty, her skill in dealing with critical cases, and the ability with which she has managed the domestic economy of the hospital. The balance sheet of the combined institution shows a profit of over £14.

### SHORT ITEMS.

We learn that the committee of the Maidenhead Cottage Hospital have not yet come to any decision as to how the £10,000 given by Mr. Astor shall be applied. At present the hospital only contains 12 beds.—Sir Henry Harben will open the new balconies which have been erected at the North London Hospital for Consumption, Hampstead, on Wednesday, June 20th, at three o'clock.—Miss Vaughan, the new matron of the Royal Bucks Hospital, asks us to state that she was not sister at the General Hospital, Wolverhampton.

### Lectures on Nursing for Probationers.

By E. MacDowel Cosgrave, M.D., &c., Lecturer to the Dublin Metropolitan Technical School for Nurses.

### VIII.—THE BLOOD-VESSELS.

There are three classes of blood-vessels in the body—arteries, capillaries, and veins. The arteries carry pure blood to the capillaries, which are in all the tissues of the body; when the blood has become impure it is carried by the veins to the lungs to be purified.

Fig. 14.

The Arteries of the Upper Extremity.

The arteries have strong muscular walls; they are deeply situated chiefly along the bones, and the blood passes through them in pulsations caused by the beating of the heart. The veins are more numerous, some are deep, running alongside the arteries, others are near the surface just under the skin. They have valves which prevent the blood flowing the wrong way. The blood is helped up through the veins by the pressure of the muscles; thus varicose veins are relieved by exercise, and made worse by standing still. The capillaries are very small, requiring a microscope to enable them to be seen. Their walls are so thin that the nourishment and oxygen can easily pass out, and the wornout tissues and carbonic acid gas can pass in.

The blood leaves the heart by the aorta, which passes up from the heart, crosses over it, and runs down the front of the spine. It gives off branches to supply the upper parts of the body. The carotid artery runs up the side of the neck. The subclavian runs under the clavicles into the armpit, where it is called the axillary, and down the arm beside the humerus, where it is called the brachial; at the elbow the brachial splits into the radial and ulnar, which run down the bones of the forearm.

The intercostal arteries run between the ribs and supply the walls of the body. Arteries supply also the tissues of the heart and lungs, and the digestive canal and other abdominal organs. At the level of the umbilicus the aorta divides into two branches, these give off the external iliac which runs to the top of the thigh; here it passes into the femoral, which runs down the thigh to the inside, and round to the back of the knee.

The pulmonary arteries and veins are an exception to the usual rule; the former carry impure blood to the lungs, the latter carry pure blood back to the heart.

When an artery is cut the blood comes out in jets; it is of a bright red colour and is flowing from the heart.

When the vein is cut the blood issues in a steady flow; it is of a dark purple colour and is flowing towards the heart. When capillaries are cut there is only oozing of blood, which is generally bright coloured.

The natural manner in which hæmorrhage is stopped is by coagulation or clotting. This does not take place in injured vessels, but does when blood meets the air. If hæmorrhage is rapid there is not time for clotting to take place, but gradually, as more and more blood is lost, the sufferer gets fainter and fainter, which makes the action of the heart slower and weaker, until the blood-stream becomes so feeble that clotting can take place. So in hæmorrhage the patient ought to be kept quiet and not stimulated.

The simple means of arresting hæmorrhage are cold, pressure, and elevating the part; these measures lessen the blood-stream, contract the vessels, and give time for clotting.

So a slight cut should be sponged with cold water, a pad of lint, either dry or wrung out of cold water, placed on it, and this should be secured by a firm bandage, and, if the cut is on a limb, the part should be raised.

In venous hæmorrhage the patient should be placed lying down, a firm pad should be tightly bandaged over the cut, and, if that is not sufficient, another pad should be bandaged on the vein at the side of the cut farthest from the heart.

The veins of the calf of the leg often become enlarged or varicose; these veins sometimes burst, especially if there is an ulcer in the neighbourhood, for this removes the support of the skin. In such a case the patient should be put lying down with the leg well raised, and the place should be bandaged.

Arterial hæmorrhage is sometimes very hard to check, even firm pressure failing. A graduated pad—that is one small where it comes in contact with the wound and gradually getting larger until it is under the bandage—should be used; this increases the pressure on the wound. Pressure higher up on the artery helps, as it lessens the flow of blood to the wound; there are several "pressure" spots where the walls of the arteries can be compressed together, and so the entire blood-stream for the time be checked. These pressure spots occur where the artery passes over a bone and is not too deep. One of these is on the brachial artery, about two-thirds up the arm, in the groove at the

Fig. 15.
The Arteries of the Lower Extremity.

inside behind the biceps. (Fig. 14.) The radial and ulnar arteries can be compressed above the wrist, and the digital arteries at the sides of the fingers. The subclavian artery, when it passes over the first rib, also can be compressed; but in most injuries of the upper limb pressure on the brachial artery is easiest.

In the lower limb the femoral artery can be compressed at the middle of the top of the thigh; it can also be compressed about a third of the way down the thigh on a line from the middle of the top of the thigh to the inside of the knee. (Fig. 15.) Behind the inner ankle joint is another good pressure spot. This pressure away from the wound is advisable in cuts from broken glass, as pressure on the cut is dangerous if the fragments of glass have not been removed.

If pressure has to be kept up for long a tourniquet should be used. This may be improvised by loosely tying a folded handkerchief, containing a stone, cork, or other hard object, round the limb, taking care that the hard object is exactly over the artery. A stick is then inserted through the other side of the loop of handkerchief, and twisted; this tightens the handkerchief, bringing pressure to bear on the artery.

### Nursing in Ladysmith.

### A CHAT WITH MRS. LUDLOW, FORMERLY MATRON OF THE ROYAL FREE HOSPITAL.

By a Special Correspondent.

A good deal has been said and written about the bravery of the troops during the Ladysmith siege, and the British public has been enthusiastically expressing its views about their courage and endurance; but perhaps we do not all realise how much praise is due to the nursing sisters who nursed the wounded. I have just had the good fortune to see Mrs. Ludlow, the wife of Major Ludlow, who was on Sir George White's staff, and from her have heard something of what the siege meant for the nurses.

### The Netley Sisters.

"I took charge of the Town Hall during the early part of the shelling," Mrs. Ludlow said, "and then when the work became so heavy that I could not possibly do it all, I was most ably helped by Sister Hill, and we two together shared the superintendence. I am particularly anxious that the work of the Netley Sisters should be fairly recognised; sometimes a nurse's work is, as it were, behind the scenes, and though a great deal depends on her, she does not always get her due meed of praise. For instance, Miss Bond, A. N. Service, who was in charge of the operating theatre attached to the Town Hall, worked tremendously hard, and the recovery of many of the patients was due to her unremitting attention. She joined my tent at Intombi Camp, and had been nine years in the service. Then there was Sister Dowse; she was the sister superintendent, and took charge of the officers' wards in Ladysmith. After we got out to the neutral camp at Intombi she was seized with enteric fever, and after a relapse was sent, when the line of communication was reopened, to the hospital ships to recruit. She was a great loss to the nursing staff, especially as our number was so small. Her place was taken by Miss Noble, of the Army Nursing Service, who worked hard for everyone and was extremely popular, never sparing herself in the least, and doing the same hard work as the rest."

### A Boer Patient.

"Besides those I have mentioned," Mrs. Ludlow continued, "I was ably assisted by Sisters Borlais and Lees and others. The nursing staff suffered very much from fever from the first, and when the siege was raised we were only five workers out of a staff of 18 sisters. I was fortunate only to be laid up a week with inflammation of the ankle joints through being so constantly on my feet. During the few days I was ill one of my Boer patients sent me a pot of jam, which, as he was a Boer, and as jam was then 12s. 6d. a pot (when it could be obtained), was, to say the least, a delicate attention. He was a most grateful person, and usually showed his appreciation of any kindness by kissing my hand. When I gave him his share of Lady White's present of clothing, he was so delighted that I could not persuade him to take them off, not even his knitted cap, and he slept in them, and, in fact, lived in them; and when the relief came in and I broke it most gently that General Buller was in Ladysmith, he said, 'Plenty of scoff now, Sister'—meaning that he would now get plenty of food."

### Details of the Siege.

"I was summoned," said Mrs. Ludlow, "on October 13th, to take charge of the Station Hospital at Maritzburg, to enable the Netley sisters to come to Ladysmith. I had only been there five days when I was sent with four other sisters to Ladysmith in anticipation of the Elands Laagte fight on October 21st. We arrived, after travelling all night, early on the Saturday morning, and on Saturday night our poor wounded came in. There were 40 beds in the Town Hall, and the wounded came in in crowds. It was such a sickening sight; and all Saturday night and Sunday they came in quicker than we could find places for them, every case seeming worse than the last. The floor was covered with wounded, dying, and dead, and we could only get to our patients by stepping over them. The beds were only enough to accommodate the most hopeless cases. I can never forget the sight; and many a brave soldier died there as bravely as he had fought, with never a murmur except an occasional groan of pain or expression of gratitude for what had been done to ease his suffering. The Gordon Highlanders suffered terribly; every second man seemed to be a Gordon; the kilts, unfortunately, were a distinctive mark in the field."

### Twenty Thousand Boers.

"Early on Monday, with our second lot of wounded from Lombard's Kop, came the first shell over Ladysmith. It was an awful day. Shells were flying all over the town—there were thirty guns on Ladysmith—and we soon filled three churches with wounded. The shock of the firing was terrible, and had an awful effect on some of the cases; indeed, it was so bad that an order was given for the town to be emptied of all except those with special permission from Government to tend the wounded, and only just in time, for next day we were cut off from the line of communication. There were 20,000 Boers surrounding us, and we never expected to come out alive, and all wrote letters of farewell to our friends. In the first forty-eight hours 1,500 shells came into Ladysmith. After a week of heavy shelling General White sent an officer under the flag of truce to ask General Joubert to allow him to move the wounded to a place of safety as the town was being so heavily bombarded, and he allowed us to remove to the Intombi Spruit just under 'Long Tom' on Bulwana Hill, the gun which did so much damage to Ladysmith. Here we remained for the four months of the siege, having no communication with the outside world whatever and surrounded by guns and Boers on all sides. Being just under the Boer camp we were virtually prisoners of war, and had to absolutely observe all the conditions laid down by General Joubert, one of which was that we were to hand over cameras and field-glasses. There were, of course, various other conditions, and if our camp at night seemed to the Boers particularly active we were reminded that we were under observation by their searchlight being turned on us."

THE LAST MONTH.

"Time went on, and General Buller did not come; we were all reduced to half, and then quarter, rations; Bovril and meat extract made from horse-flesh was all we had to give our men in the way of nourishment, with the exception of a very little rice, arrowroot, and starch. This began about a month before we were relieved, and you can imagine what it was to see our patients hungry and suffering. Our camp was by this time a large one, numbering altogether two thousand sick and wounded men. Enteric had become very prevalent, and with fresh cases arriving each day from Ladysmith our numbers soon increased with alarming rapidity, and our little cemetery of seven hundred souls reminded us sadly that life and death had been very close to each other."

A MEMENTO OF THE SIEGE.

Mrs. Ludlow showed me, as a touching little memento of the siege, a little brown loaf, a siege loaf, of mealie meal; one quarter of this was one person's share when things were worst, and the garrison was on quarter rations. "The meat consisted of the flesh of horses, mules, and trek oxen, and one tin of Nestle's milk (price 6d. in England) had to be divided among sixty sick persons. The stores were sent out from Ladysmith early every morning by train, with the few little comforts we could get for our patients. In December we had two batches of wounded in from Gun Hill and Surprise Hill. On January 6th the Wagon Hill fight took place, and the shots from the rifles rained on the tops of our tents. I had emptied all my marquees in anticipation of this fight, and I received close upon 100 of the most severely wounded, for at the time I had charge of all the surgical cases with the exception of officers and cases that were considered convalescent enough to go into tents to be looked after by orderlies. I had three weeks' hard nursing, and I am thankful to say that out of nearly 100 men I only lost six or seven cases, and these were absolutely hopeless from the first."

THE FEELING IN THE CAMP.

Finally, Mrs. Ludlow told a very touching story of the relief, how when it came at last everyone was so low and despairing that they could hardly believe the long siege was over.

"I can never describe the thankfulness and relief in camp," she said. "Men low and depressed and half-nourished got up and out of bed and dressed, and would look about and try to walk as far as possible to hear and see all that was going on. We were a little weary with the privations and suspense, but our call to duty found us ready to take up whatever fresh work lay to hand, like the brave soldiers we tried to help."

### Resignation.

BEDFORDSHIRE HOSPITAL TRAINED NURSES' INSTITUTE.—To the great regret of the staff, Mrs. Rawson has resigned the position of honorary lady superintendent of the Bedfordshire Hospital Trained Nurses' Institute, after 11½ years' service, in consequence of ill-health, at her doctor's advice. Mrs. Rawson was trained at the Middlesex Hospital. She held the post of sister of the Lind Gallery, Brompton Consumptive Hospital, from 1886 to 1887. Since then she has done private nursing, both on the Continent and in England. In 1889 she started the Nurses' Institute, St. Peter's, for the doctors in Bedford, beginning the home with three nurses. It increased so much, however, that the accommodation became too limited. After anxious thought she asked the Duchess (Adeline) of Bedford if she would open a bazaar in aid of funds for an enlarged building, which she kindly did, and after a two days' sale the handsome sum of £621 was realised. The staff at the institute consisted of 25 in 1899.

### Dispatch of Sixty Nurses to South Africa.

THE exodus of nurses to the front continues. Last Saturday no less than twenty-five, in addition to those mentioned in our last issue, embarked, and on Saturday next thirty-five more members of the Army Nursing Service Reserve will leave England for South Africa. The names of the sixty nurses are as follows: Miss M. Atkinson, Miss E. M. Bankes, Miss E. M. Beesby, Miss R. D. Bowhill, Miss J. Butler, Miss A. F. Byers, Miss A. R. Callum, Miss J. Caldcleugh, Miss K. M. Carter, Miss F. H. W. Carver, Miss E L. Corser, Miss J. Crosby, Miss K. M. Despard, Miss E. Ellis, Miss A. W. Fraser, Miss F. E. Filkin, Miss F. L. Gledhill, Miss M. Goldsmith, Miss E. M. Halliwell, Miss C. Hamilton, Miss L. Hand, Miss A. F. Hobbs, Miss B. E. Hutchison, Miss E. W. Jaynes, Miss A. B. Joel, Miss A. F. Langshaw, Miss C. Lamont, Miss E. M. Lendon, Miss K. E. Luard, Miss M. May, Miss J. Mitchell, Miss M. J. Mill, Miss L. M. Monk, Miss A. Murton, Miss A. Myring, Miss L. C. M. Noble, Miss M. L. Pick, Miss A. M. Poulter, Miss E. Prangley, Miss A. J. Rees, Miss D. I. Richards, Miss A. Ridley, Miss L. Sands, Miss E. A. Scantlebury, Miss L. E. Snape, Miss S. Smith, Miss J. Stephenson, Miss G. H. Swanton, Miss H. Suart, Miss J. K. Szezepaneka, Miss E. Tait, Miss R. E. Tarbuck, Miss E. Taylor, Miss J. J. Walworth, Miss M. Walker, Miss J. Ward, Miss M. Warren-Smith, Miss A. J. Webb, Miss E. M. Wilson, and Miss E. M. Woodman.

MISS E. M. BANKES was trained at the Devon and Exeter Hospital. She afterwards worked on the private staff there, and subsequently was staff nurse in some of the male wards. Last year she took the temporary post of staff nurse at the Smallwood Hospital, Redditch, and was there over six months. Since September she has been charge nurse at the Grove Hospital, Lower Tooting, and has had charge of the diphtheria and enteric wards.

MISS JANET BUTLER was trained at the Royal Free Hospital for three years, and has since been engaged in private nursing.

MISS LOUISA HAND was trained at Warneford Hospital, Leamington, for three years, and was subsequently staff nurse. Since October, 1897, she has been engaged in private nursing.

MISS ADA MYRING was trained at St. George's Infirmary, Fulham Road, and at the City of London Hospital for maternity work. She has since been charge nurse at the Grantham Hospital and County Hospital, Huntingdon. Since last July she has been attached to the Bournemouth Co-operation of Trained Nurses.

MISS EMILY ANN SCANTLEBURY was trained for three years at the Royal Hospital, Portsmouth, and was subsequently staff nurse. Since May, 1893, she has been attached to the Nightingale Home, Southsea.

MISS HANNAH SUART was trained for three years at Guy's Hospital, where she has been staff nurse since July, 1898. She holds the L.O.S. certificate.

MISS M. M. WALKER was trained at Belvedere Fever Hospital, and the Royal Infirmary, Glasgow.

MISS ELIZABETH MARGARET WILSON was trained at Grimsby Hospital, and since May, 1895, has been engaged in private nursing.

### Wants and Workers.

WILL readers kindly let me know of places where the Board of Guardians contributes towards district nursing expenses? Penrith and Llandaff are known to me. Address Miss Brooke-Hunt, Merton Grange, Slough.

### Nursing at the Imperial Yeomanry Hospital.

### REMARKABLE PROGRESS.

WRITING from Deelfontein under date of May 14th, a member of the Army Nursing Service Reserve says: In six weeks the Imperial Yeomanry Hospital has grown wonderfully. It is now nearing completion, and is certainly a most imposing-looking place, with its rows of tents and huts, and its well laid-out roads, bordered with whitewashed stones. Even to the most casual observer the growth of a camp like ours must have some interest; but to people who are acquainted with the working of hospitals in all their many details—hospitals that are the outcome of years of thought and labour—this is indeed little short of a marvel. When one remembers that less than three months ago Deelfontein was merely a station and a store, a watering-place for trains in the middle of the vast Karroo, and that now it is the site of a thoroughly well-appointed and equipped hospital of some 700 beds, dependent on itself for almost all its own comforts and necessaries, it is surely a thing to wonder at. Owing to the large number of cases of enteric at Bloemfontein, De Aar, and other places, the authorities here have already found it necessary to give up four wards, each holding 20 to 24 beds, for the nursing of acute cases.

### THE SADDEST CASES.

Convalescent patients are as soon as possible moved to tents, and it always seems to me that these wards are the saddest in the whole hospital. Practically all the patients in them have fought bravely for Queen and country at Belmont, Graspan, Paardeburg, &c., have endured all the hardships of the campaign, and have escaped the dangers of the battle-field unhurt, only to fall victims to a more deadly enemy still. One man I knew of—doubtless there are many others—had been wounded, and had quite recovered, had also been recommended for the V.C., only, alas, to die of typhoid fever. Still even these wards are very far from being altogether sad, for it is wonderful to see how quickly the men recover from severe attacks when they once make a start, and how hungry, bright, and happy they are! These four wards are all placed together, with their offices and belongings alongside, so that, as far as possible, enteric cases are isolated.

### A WARD FOR OFFICERS.

Next worthy of note is the Sherwood Rangers Ward, which is one of six beautiful English-made huts, and is specially set apart for the use of officers. In a hospital where everything is new and up-to-date comparisons are obviously absurd, but certainly in this, as in every other ward in the camp, everything that is possible is done for the comfort and happiness of the patients. Another ward is allotted, as far as is practicable, to the special use of yeomen; otherwise the hospital, though theirs in name, is open to any man wearing the Queen's uniform, whether colonial, volunteer, or regular. The operating theatre is certainly worth a visit. Of course, it has not the marble walls of some of our newest theatres at home, but a cleaner, lighter, better-equipped little place it is impossible to imagine. All the instruments and appliances that can in any case be wanted are here, and the $x$ rays have already been of the very greatest use in locating bullets and showing splintered bones, thus materially helping the surgeons to do the best that could be done for the patients.

### THE RECREATION TENT.

Passing on from the theatre we come to the recreation tent, which is well supplied with comfortable chairs, games, and papers of every description; the latter are particularly appreciated—how much, it is almost impossible for people at home to realise, for though, comparatively speaking, the Yeomanry Hospital is so near the seat of war, yet there is a veritable dearth of news, and it is hardly too much to say that we in South Africa know nothing of advances, losses, and victories till London has almost forgotten them. Rumours we hear, it is true; the guard of some passing train, or perchance a new patient, tells us what he has heard—but facts! The best part of the recreation tent is its lending library, and this is in truth a grand institution. There are a great number of books of all sorts and descriptions, many of them being standard works, besides the latest novels. This library vastly lessens the tedium of Tommy's convalescence, and certainly adds much to his pleasure in life; to change a book for a comrade who is tied to his bed makes a nice little morning stroll for the more fortunate friend.

### THE CHAPEL CHANCEL AND THE DENTIST'S CHAIR.

Opening out of the recreation tent, but partitioned off during the week, is the chapel chancel, the recreation tent itself being converted on Sundays into the body of the church. Much care and energy have been and is being spent on this little chapel, and it is only necessary to be present either at the hearty Sunday morning parade service or at evensong to realise how fully the whole population of the camp appreciates its privileges in having such services and a resident chaplain. From the chapel we pass on to the dental surgery and dispensary, both these essential departments of a hospital being as well stocked in all their details as they possibly can be, and certainly many a soldier has gone away from the discomfort of the dentist's chair better able to deal with ration biscuits, which, as everyone knows, are very excellent, but not particularly soft.

### THE EXCITING EVENTS.

Of course the arrival of a Red Cross train or the departure of convalescents for Cape Town are great events in the life of the camp. The men who come down by the former are very fortunate in each getting a present from the Cape of Good Hope and British Red Cross Societies of a bag, containing a towel, piece of soap, sponge and bag, tooth brush, suit of pyjamas, and slippers! Veritable lucky bags they are, as Tommy himself calls them, and of all the useful things that have been sent out for him none have been more gladly welcomed or added more to his comfort than these bags.

### THE LAUNDRY.

An account of the hospital would certainly be incomplete without a few words about the laundry. This is now practically in full working order, and is set up with machinery of the most modern and approved type. It has of necessity been not the least expensive part of the hospital, but, like everything else in the camp at Deelfontein, reflects the very greatest credit on the organisers of the Imperial Yeomanry Hospital.

### To Nurses.

WE invite contributions from any of our readers, and shall be glad to pay for "Notes on News from the Nursing World," or for articles describing nursing experiences, or dealing with any nursing question from an original point of view. The minimum payment for contributions is 5s., but we welcome interesting contributions of a column, or a page, in length. It may be added that notices of entertainments, presentations, and deaths are not paid for, but, of course, we are always glad to receive them. All rejected manuscripts are returned in due course, and all payments for manuscripts used are made as early as possible at the beginning of each quarter.

### Nursing in Tuberculous Diseases of Joints.

Clinical Lecture delivered to Nurses at the City Orthopædic Hospital, by NOBLE SMITH, F.R.C.S.Ed., Surgeon to the Hospital.

### BED-SORES.

IN orthopædic cases bed-sores are occasionally a trouble, but they are far more capable of management, or prevention, than when the spinal cord is seriously or permanently damaged. Even in severe caries, when paralysis may have occurred from pressure or extension of inflammation from the diseased bones to the spinal cord, bed-sores ought, as a rule, to be prevented, or if they occur they ought to be easily controlled. The paralysis is temporary and should pass away with efficient treatment. I have known patients paralysed entirely as to motion from the waist downwards, including paralysis of the sphincters of the bladder and the rectum recover perfectly from the paralysis without the formation of any bed-sores. With the object of preventing such sores, the following points are to be borne in mind: Great cleanliness, smoothness of sheets, dryness, sponging, and powdering, keeping the bed free from crumbs, skilful arrangement of pillows, timely use of air cushions, application of various alcoholic preparations and collodion and other substances to the skin. It is always desirable in these cases to shift the patient frequently so that no one part gets pressed upon for long at a time. In making these movements you must be careful to avoid undue disturbance of the diseased parts of the spine. Whilst on this subject of pressure sores we may consider that of irritation, which sometimes takes place from the use of apparatus. I may say at once that no sore ought ever to take place from such a cause. Some irritation may be produced at times from want of adjustment of the apparatus, whatever that apparatus may be, and this applies to all kinds of splints, including those used in fractures. As far as the nurse is concerned, it is her duty to intimate to the surgeon any sign of such irritation.

All the apparatus used in this hospital are either made of wood or pliable steel. Steel is the lightest substance that can be used, as it can be made thinner than any other metal, and it has properties of elasticity peculiar to itself. It can be so tempered that the surgeon can bend it in any position he wishes, and a very little alteration will relieve a part from undue pressure. With some patients the skin is so sensitive and tender that very slight friction will cause irritation, and under such circumstances applications of various substances (the best of which are spirituous solutions of tannin or Friar's balsam), should be painted on the skin every day at the places where the friction occurs. This extreme sensitiveness is generally a sign of general weakness, and when the patients become stronger the irritability usually passes off.

The application of adhesive plaster to the skin, as for instance when used for applying weight extension to a limb, may cause irritation or abrasion of the skin. In the case of the lower extremities, gaiters, made of stout holland fitting closely to the leg, are much more comfortable to the patient than strapping, and pressure is thus avoided. In cases where we have to extend the hip-joint it is desirable to carry the gaiter above the knee so as to take some bearing on the lower end of the thigh as well as the leg, otherwise too much strain is placed on the knee-joint.

Although the surgeon generally decides upon these points it is nevertheless the duty of the nurse to watch as to the results of treatment, and to bring to the notice of the surgeon any discomfort felt by the patient, and I personally hold that the nurse should make suggestions to the surgeon if anything occurs to her which she thinks might help the patient.

There are two main faults which are possible on the part of a nurse—one is indifference as to her work, a fault which, in my experience is not common, the other is too much enthusiasm. The latter may mean undue interference in work which belongs to the surgeon. The correct course is for the nurse to be ever watchful, ever resourceful, always ready to refer to the surgeon in matters of doubt. Mistakes may be made by any of us, and a nurse, if she thinks she has made a mistake, should lose no time in acquainting the surgeon with the fact. To explain what I mean, I will refer to one not infrequent error. I mean the placing of hot-water bottles in contact with a patient after an operation. I have never known such a mistake made at this hospital, but I have known it made by nurses who were supposed to be thoroughly trained. Blisters may be easily set up in this way, and if such a thing happens, the surgeon should be told of it as soon as possible. I have known several instances in which a nurse has tried to hide the matter and treat the wound herself. This, in one case, led to very serious results.

### Minor Appointments.

ORIOLET HOSPITAL, LOUGHTON.—Miss Edyth Davis has been appointed Sister in Charge. She was trained at West Ham Hospital, and has since been sister at the Children's Hospital, Belfast; superintendent at the Children's Home Hospital, Sheffield; and temporary deputy matron of the West Ham Hospital.

BIRMINGHAM AND MIDLAND COUNTIES' TRAINING INSTITUTION FOR NURSES.—Miss H. A. Quinton has been appointed Home Sister. She was trained at the North Staffordshire Infirmary, Stoke-upon-Trent, for three years, where she was afterwards charge nurse for two and a-half years.

INFECTIOUS DISEASES HOSPITAL, TOLWORTH.—Miss Hannah D. Freeman has been appointed Superintendent Nurse. She was trained at the Southern Hospital, Liverpool, and has since been nurse in the Acland Home, Oxford, and under matron at the Infectious Hospital, Bicester.

GROVE FEVER HOSPITAL.—Miss Louise Mary Biggs has been appointed Superintendent of Night Nurses. She was trained at the Western Infirmary, Glasgow, and has been charge nurse at the Fountain Hospital, Lower Tooting.

BIRMINGHAM GENERAL HOSPITAL.—Miss A. Baker has been appointed Sister. She was trained at Leeds Royal Infirmary, and was subsequently sister, first at Bolton Infirmary, and then at Cardiff Infirmary.

WOLVERHAMPTON GENERAL HOSPITAL.—Miss Maude Sawle has been appointed Sister. She was trained at the Cardiff Infirmary, and has since been sister at the County Hospital, Newport, Monmouthshire.

FOUNTAIN FEVER HOSPITAL, LOWER TOOTING.—Miss Annie Gillespie has been appointed Charge Nurse. She was trained at the Royal Portsmouth Hospital for three years.

ST. OLAVE'S INFIRMARY, ROTHERHITHE.—Miss Minnie Burrell has been appointed Charge Nurse. She was trained at St. Olave's Infirmary, and has since been nurse.

### Presentation.

GROVE HOSPITAL, LOWER TOOTING.—On the occasion of the departure of Miss Dacher from the Grove Hospital, Lower Tooting, where she has been acting matron since the opening (a period of nine months), a pleasing little ceremony took place. Dr. Beggs, the medical superintendent, presented her, on behalf of the medical and nursing staff, with a silver Queen Anne tea service; from the domestic staff, a gold curb bracelet and brooch; and from the male staff, a pair of ivory hair brushes. Miss Dacher takes with her on resuming her own duties as matron of the hospital ships, the good wishes of the entire staff, to whom she has endeared herself by her unfailing kindness during her temporary residence at the Grove.

### Some Difficulties of a Matron's Life.

By a Matron.

"For every evil under the sun
There is a remedy, or there is none.
If there's a remedy try to find it,
If there's no remedy, try not to mind it."

The old nursery rhyme recurred to my thoughts as I pondered over a certain evil, or rather difficulty, connected with hospital life, and speculated as to its causes and remedy. The difficulty in question is this, that a matron of a fairly large hospital knows so little of her nursing staff, and, for this lack of knowledge, can give them neither the sympathy, help, nor instruction they so much need.

How can she get to know them? I do not speak of small cottage hospitals, where the matron works hand-in-hand with her one or two nurses, nor of slightly larger ones, where she occupies more the position that a sister has in a big London hospital; but one, for instance, of about 60 beds, with a nursing staff of between 20 and 30. She interviews candidates, who are on their very best behaviour for the time being, and, as has been remarked in a recent book on nursing, "In an interview of a few minutes is supposed to discriminate by some fine intuitive process between the fit and unfit." Well, she does it as best she can, and in a hospital of the size mentioned, the choosing of probationers is apt to fall entirely to her. She asks them certain stereotyped questions, to which they return more or less stereotyped answers, and by-and-bye they are admitted on trial. What then? They are straightway put into one of the wards under a sister or staff nurse, and from them, generally, and not from their own observation at all, they derive their most permanent impressions of the matron. Usually, they learn to fear her as a person possessed of some weird and occult power chiefly of doing them harm, and liable to be called into action by the most trivial fault on their part.

### Matron and Probationers.

How much did we see of our matron when we were probationers? Was it not only at certain meals, at prayers, or once a day perhaps during her periodical visits to the wards, or on those, it is to be hoped, rarer occasions when we were "sent for" to receive reproof or criticism of our work or conduct? I remember in our training school we looked upon the matron chiefly as a person to be dreaded—a mighty potentate, whose principal function in life was to find out our weak points; who, in a lesser degree, it is true, had power to grant certain favours, such as extension of leave, or an order for new uniform, but who, on the whole, was clearly a functionary to be avoided as far as possible, and who, when she had to be approached, must be conciliated by immaculate uniform, well-chosen phrases, and scrupulous official courtesy. We should as soon have thought of unburdening our hearts to the Secretary of State as of telling our matron any of our home troubles or ward difficulties, or of seeking her on any occasion when we needed sympathy and encouragement. The only time we ever went to consult her was when we were leaving and wanted to obtain a post elsewhere, and it was generally then, and not till then, that we made the discovery how much she had our welfare really at heart. We found what infinite losers we had been all this time by not making more of a friend of her, and profiting by the advice and help she would have been only too glad to have given us had we not held ourselves so aloof from her.

### The Matron's Office.

Unfortunately, almost the chief, though happily not the whole, duty of a matron does consist of reproof and criticism. Her office is invariably the cave of Adullam for the hospital, and into it are brought unceasingly all the multifarious sins, negligences, and ignorances that can by any possibility occur on the premises in the course of twenty-four hours. She is besieged all day long by various applicants. Friends of patients apply to her for beds, clothes, money, surgical appliances, tickets for convalescent homes, bath chairs, water beds, district nurses, private nurses, and what not. The housekeeper comes with weird tales of bursting boilers, leaking cisterns, froward maids, recreant tradespeople, and these two last cover an immense deal of ground and include a very large category of complaints. The servants, themselves, have many grievances. They want more money, more liberty, or to change their off duty hours with someone else. Parcels, letters, telegrams, visitors, all converge to the matron's office as to one common centre. Members of the nursing staff come at all hours and for all conceivable things. The matron takes her breakfast while listening to the night superintendent's report, and her tea to a running accompaniment of taps at the door. She sorts out the letters that come by the morning post, preparatory to taking them round the wards, and before she can even read the addresses a message comes that the whitewashers want to know where they are to begin work, that the man has brought the new crutches, and will she tell him if they are the right shape, &c., &c.

### The Temper of an Archangel Needed.

It is simply astonishing too the ingenious way in which things get lost, broken, or spoiled, or in some way out of order, if it is at all necessary to keep the expenses down and count the cost. The worry of knowing that the bills for housekeeping, dressings, &c., are much higher this month than last, the knowledge that waste is going on in quarters where she is powerless to prevent it, and the consciousness that while she is attending to one thing she is perforce neglecting another is enough to try the temper of an archangel. In the hospital I have in my mind just now the matron has to prepare all the sterilised and cyanide gauze that is used, and to keep all the instruments in order herself. She gives out all the stores, admits and writes admission papers for all the patients, and goes round the wards with the visiting staff.

Many candidates also apply for admission, and this gives a good deal of work, both in correspondence and interviews. From the schoolgirl of sixteen, who "has always wanted to be a nurse," to the mature woman past the mysterious bourne of forty years—that tyrannical age limit about which we have heard so much lately—and who, however capable she may appear, yet cannot be allowed to enter our ranks as a beginner, although she assures us pathetically that she is "quite strong, and willing to do anything," they all want information, explanations, advice, and counsel, and all these things take time. There is the carving to be done at the nurses' dinner-table, there is the supper to serve, and prayers to read. Members of the Visiting Committee come at all manner of times and seasons, and require to be taken, aye, and taken smilingly too, round the wards.

There are books and registers and lists to be kept. There are uniforms to be attended to, there is the household linen to be looked after, there are sick members of the staff to be nursed, holidays to be arranged for, lectures to be attended, and the nurses kept up to their work in every particular. Being a hospital which does not offer any special inducement to its nurses to stay on after the completion of their three years' training, most of them go at the end of their time, thus leaving the wards always in the hands of comparative juniors, which necessarily increases the responsibilities of the matron.

In the midst of all these duties, which, of course, include a far greater variety than could possibly be enumerated here, how, I repeat, is a matron to get to know much of her nursing staff? The duties are perfectly legitimate and must be done, but they leave little time for anything else, and during the doing of them an earnest-minded woman, who feels her responsibilities, is constantly wondering how she can adequately fulfil what she feels to be her first duty—that to her nursing staff. If she goes into their sitting-room and sees a group of nurses laughing and chatting together, what will she find? No matter how good her motives, how pleasant and kindly her manner, the laughter and cheerful talk will most likely give place to a confused silence on her entrance, broken, perhaps, by some conventional remark from the more self-possessed of the party. The rest will probably subside into a schoolgirl giggle, blush as if they had been detected in some wrong-doing, and seize the earliest opportunity for edging out of the room.

### Plans that Failed.

One matron I know of tried the plan of inviting her nurses to afternoon tea with her once a week, and another used to have quite a big "At Home" for them now and then. Neither of these plans, as far as I remember, was a great success. An unaccountable and stony silence seemed to take possession of us as soon as we got inside the door, and a wild desire to escape was the one emotion we were conscious of.

Of course a certain amount of what may be called officialism is necessary in a hospital, and must be kept up, if discipline is to be maintained at all. The matron who would laugh and joke with her nurses and permit them at any time to treat her without the respect due to her official position would find herself nonplussed when she had to find fault with the same people.

Yet she wants to get to know them, to know them each personally, not only officially, if she would be the help to them she rightly felt she ought to be; and where all are so busy this is not easy. Even if a nurse were disposed to come of her own accord to the matron to consult her about something, she is often hindered by quite legitimate reasons. She may not like to interrupt the busy chief after a hard day's work at a time when she knows she is alone in her sitting-room for fear of bothering her should she be resting. That reason, though it has its origin in a praiseworthy and unselfish motive, I think may safely be set aside. I believe there are very few matrons who, however tired they might be feeling, yet would not welcome an interview of this kind. Again a nurse may be hindered by her companions, who are sometimes too ready to suspect favouritism or tale-bearing, and who would look with suspicion on one of their number who was known to be too friendly with the authorities. And this second reason also often prevents a faithful report being given by head nurses of a probationer's work.

If a matron asks questions about her conduct and work she only gets from the head nurse generalities by way of reply. She, perhaps, feels vaguely that something is being kept back, but cannot find out what it is, and after a month's trial sometimes passes an unsatisfactory probationer for want of an adequate reason for refusing her.

Patients cannot for obvious reasons be questioned. Doctors often have their favourites. Besides, it does not by any means follow that because a nurse can succeed in pleasing a doctor that therefore she is a good and desirable person in all other ways. Indeed, the reverse is sometimes true. So that after all is said and done, and thought and puzzled over, the matron with a sigh has to fall back again upon her own resources, and make the best of what seems an impossible task.

### Her Need of Help.

And of all people she seems to have the fewest to help her in her difficulties. Books for the instruction of probationers have been multiplied. They are positively surfeited with them—all most excellent—besides being pursued throughout their hospital course by lectures, classes, examinations, and the various helpful articles that appear from time to time in the different nursing journals. Staff nurses have their ward sisters to fall back upon, ward sisters the matron, and indeed, she requires to be a very rock of strength to meet all the demands made upon her. She is supposed to know everything, attend to everything, and is responsible for everything in the domestic and nursing departments of the hospital. Whoever does wrong, she never must. Very little allowance is ever made for her. In some places she is practically never off duty. And she must never fail in temper, tact, nor judgment. The "fierce light that beats upon a throne" beats upon her in a lesser degree, and no St. Simon Stylites, shivering on his pillar, can be made the subject of more criticism and observation than she is.

### The Evolution of the All-round Ideal Matron.

Truly her difficulties are many, and often they are increased unconsciously by her nurses, who persistently hold themselves aloof, and who forget how much each one of them can do towards lessening existing evils, and causing the wheels to go round more smoothly, by meeting her half way in her plans for their benefit, by suggesting and stating fearlessly what they would like and what they feel is needed in their hospital for its improvement, and, above all, by being entirely loyal on all occasions. The absolutely perfect hospital has yet to be built, the wholly immaculate nursing staff yet to be organised, and the all-round ideal matron yet to be evolved. But we all want to help on the evolution.

### The Medical, Surgical, and Hygienic Exhibition.

Nurses who have visited the Medical, Surgical, and Hygienic Exhibition at Queen's Hall will have found much to interest them from a professional point of view. Passing by the more strictly surgical appliances on the instrument makers' stalls, which are dealt with elsewhere, a few of the exhibits deserve a word of mention. The "Queen's Nurses'" District Bag, with interchangeable, washing linings, and specially designed polished wood stand for bottles, measures, &c., shown by W. K. Stacey, for instance, is an admirably useful article, and the aseptic wallets, chatelaines, and other nursing requisites, also to be seen on this stall, attracted much favourable comment. On Mrs. Ballin's stall, well supplied with useful literature on nursery subjects, and specimens of the practical baby clothing which is proving so great a boon to mothers and nurses, was an ingenious apparatus for "humanising" milk, designed by a medical man for domestic use. The "Lupa Humaniser" ought to become very popular, by simplifying the proper home preparation of cow's milk for babies in a most satisfactory manner. Nursing books were displayed in attractive abundance on the "Scientific Press" stall. Nurses who have tested the excellences of the Bella-Wattee teapots will have been glad to notice that these are now to be had in the favourite brown ware, and that the latest design is "spoutless," whereby the risk of breakage is greatly lessened. Messrs. Southall's stall is always worth a visit; their "cycle basket" for district nurses, designed by a nurse, is excellent for its purpose, and neat and workmanlike in appearance; their various sanitary appliances called as usual for nothing but praise, and especially to be commended is the "Midwife's and Nurse's Chart and Case Book." Altogether the exhibition of 1900 merits very favourable comment.

### Echoes from the Outside World.

### AN OPEN LETTER TO A HOSPITAL NURSE.

Lord Rosslyn's message last week was only a little too previous. In his joy at obtaining his liberty, and his desire to forestall everybody else, he conveyed by his startling telegram the impression that Pretoria had fallen because President Kruger, with all the gold he could seize, had taken himself off. That the place was in a state of panic, numbers fleeing because they believed the triumphant British would show no mercy, seems to have been true enough, but a rumour also quickly followed that Pretoria meant to fight, and that 10,000 Boers were outside the town to dispute our entrance. It created no terror, however, in our minds, and then on Tuesday came the good news from Lord Roberts himself. "We are now in possession of Pretoria. The official entry will be made at two o'clock this afternoon." So few words, and yet it meant so much to us all! My first thought was of the four thousand odd prisoners who had been waiting so many weary months for the sound of the tramp of friendly feet, and the certainty of liberty once more. It was good to read that General French with some cavalry had been sent to set the captives free immediately following the arrival of Lord Roberts. Mrs. Kruger and Mrs. Botha have been left behind by their respective husbands.

Does it not seem strange that the National Gallery is not properly protected against fire? Last week it was within touch of conflagration owing to a fire breaking out in a house adjoining the western end. Those who have explored the scene assert that if a south-westerly gale had prevailed, instead of an east wind, incalculable damage might have been done, even if the national treasure-house with its priceless contents had not been burnt to the ground. The mere idea of such a calamity is enough to make one shiver; and the fact that Mr. Hawes Turner, the keeper of the Gallery, goes so far as to say that the shops and buildings which abut on it ought to be pulled down, and a street or open space provided on the west side, is certainly calculated to stimulate uneasiness. Of course, it is a question of cost, and the Treasury, perhaps, cannot be blamed for not supplying the First Commissioner of Works with the necessary money, unless the public insist upon the immediate isolation of the building. It may be cheaper for the moment to wait until a large scheme of improvement can be carried out; but if, while we were waiting, the catastrophe, which is known to be possible under existing circumstances occurred, the loss would be not only tremendous but irremediable. By a curious coincidence, the same week in which the National Gallery was threatened with loss by fire, it sustained a loss by lawsuit, and twenty pictures, bequeathed by Lady Hamilton in 1892, have been removed from the walls and handed over to the family.

Whilst our storehouse of art has been losing some of its treasures our storehouse of antiquities has been so enriched of late years that it has had to enlarge its walls. A new Babylonian room was opened to the public at the British Museum on Monday, and a great deal of a most interesting character is now so clearly and carefully arranged that he "who runs may read." There is a wonderful series of large documentary clay tablets, upon which are written records of legal and business transactions which took place 2,500 years before the birth of Christ. These records, which show the number of cows, oxen, asses, &c., given in tithe payment to the temples of South Chaldea and redeemed by money, are as fresh as if only just inscribed, and the methodical manner in which the accounts are totalled and balanced shows that even modern accountants have not learnt much more than the ancients. Also the hundred or more letters of the Babylonian King Khammurabi — another name for the Amraphel mentioned in the fourteenth chapter of Genesis—should not be missed. One is almost dumb with wonder and surprise upon reflecting that these little lumps of clay, each enclosed in its clay envelope, are the love letters of a king who lived in the time of the patriarch Abraham. Being unable to peruse them, it is impossible for the ordinary individual to know whether kings in love nearly 4,000 years ago were any wiser in the phrasing of their epistles than the more modern lover as revealed by the breach of promise actions; but one would like to know. The classification of all these Assyrian wonders has proved to the world at large that the British Museum is far above all other countries in Assyriology, a fact of which, though it intensifies our consciousness of our own ignorance, we may still be proud.

It is not many years ago since Bloomsbury was looked upon as a quarter of London only to be resorted to by those who were bound to live in town and yet could not afford to pay town prices. In those times a man who erected such a building as has just been opened by the Frederick Hotels (Limited) would have been looked upon as a maniac. But for the last half decade the tide of popularity has been steadily flowing back to Bloomsbury, till the district bids fair to be as much sought after as it was when it was one of London's suburbs. The Hotel Russell, built on one side of Russell Square, proverbially healthy with its gravelly soil, is as magnificent as any which have sprung up in the West End, and when I went over it on Wednesday evening upon the occasion of its housewarming, I found myself wondering if there was any luxury which it does not contain. The outside is built of light red brick and terra-cotta, and will stand a good deal of London smoke without detracting from its beauty; the inside is furnished by Maple and Co. in a thoroughly comfortable, as well as an artistic, manner. The reception and reading room has a large recessed fireplace with seats grouped around it, which will be most inviting on a frosty day. The dining-room is very handsome with its marble walls and marble columns, and a painting by Angelica Kauffmann over the canopied fireplace; and I also noticed some other beautiful paintings by well-known masters in the reception-room. The Winter Garden, the largest of its kind in London, is in the centre of the building, with a floor of red and white marble, Turkey carpets laid here and there, and palms in profusion. There are seven staircases provided in case of fire, and the whole building is fireproof. The bedrooms are each supplied with a Teleseme apparatus for summoning waiters, &c., and in many I observed a neatly-curtained alcove containing a bath. I have kept the best information to the end. Notwithstanding that everything conceivable is done for the comfort of visitors, and the whole place is palatial in style, the charges of the Russell Hotel are as moderate as if it were tucked away in a corner, instead of occupying one of the most central positions in the capital.

Many nurses are amateur photographers and amuse themselves with the camera while they are on their holidays. Those who are glad to take hints from a master hand at the work will thank me for recommending them to pay a visit to the rooms of the Royal Photographic Society, 66, Russell Square, where Dr. P. H. Emerson is now exhibiting a remarkably interesting collection of photographs. The progress of the amateur has been very rapid in recent years, but only an individual with genuine artistic taste could have achieved results like "A Winter Pastoral," which is perhaps the most striking study in the show. The whole of the pictures were taken by Dr. Emerson when he was on his rambles, and they can be seen any day in the week from ten to four, or on Wednesday from ten to eight. Buyers will probably be numerous.

### The Nurses' Bookshelf.

[We invite Correspondence, Criticism, Enquiries, and Notes on Books likely to interest Women and Nurses. Address, Editor, THE HOSPITAL (Nurses' Book World), 28 & 29, Southampton Street, Strand, London, W.C.]

A BRAVE POOR THING. By L. T. MEADE. (Publishers, Isbister and Co. 3s. 6d.)

THE title of L. T. Meade's new story is taken from that of a guild known as the "Guild of the Brave Poor Things," whose motto, "*Lactus sorte mea*" (Happy in my lot), was one chosen appropriately to the simple rule of cheerfulness and patient endurance under physical deformity and suffering which bound its members together. The story is a pathetic little record, and one which cannot fail to move deeply those who are in sympathy with the helpless and suffering. The guild owed its origin and its curious name to that charming book of Mrs. Ewing's, "The Story of a Short Life," in which, it will be remembered, the interest centres round a child whose great desire was to become a soldier. "Owing to an accident he was turned into a helpless cripple. By-and-by he came to learn that if he could not be a brave soldier he could at least be a brave cripple, and that the courage to bear and the courage to dare are really one and the same thing." So the guild took its motto from the motto of his house, "*Lactus sorte mea*," and the story of his life shows how bravely he tried to live up to its precept. "Let there be a book for all the wounded ones, a book of honour, a book of brave poor things, not cowardly poor things. 'It is all my own idea,' he said." Dorothea, the heroine, has a slight lameness, which entitles her to membership. She is the brave daughter of an incorrigible father, and the support of her family under difficulties before which others, less spiritually gifted, must have failed. After her working hours as a typewriter were over she joined the guild members. "Dorothea found herself facing the big room. She looked down on the faces all gazing up at her. On each one was the burden of pain, on most faces of a life-long pain. Some of those present were to have a short, sharp struggle, and then eternal rest for evermore. Some were to live long in this world, battling with the handicap which made life so difficult; but all at least now looked contented, and even interested. Every eye was fixed on the tall, slim girl who faced them." Dorothea has a romance, and a very real one, in her life, so that in spite of the sadness inseparable from the theme, the "story of the brave, poor things" is a happy and instructive one, showing how "in the shadow" lives are lived bravely and brightly, and offering many aids to reflection for those whose lives have fallen in fairer places.

A HOST OF THORNS. By HELEN COSTERTON-WILKINSON. (London: Simpkin, Marshall, Hamilton, Kent, and Co., Limited. Price 3s. 6d.)

FROM a literary point of view this story has few recommendations. The style never rises above the common-place, the punctuation is often defective, and even the spelling is not invariably accurate, though for this last defect the printer may be to blame. And yet it has a certain power, arising chiefly from the painful theme of which it treats—hereditary madness. Joan Lee had married a man in whom the taint existed—had married him, knowing of its existence. He himself had escaped it, and their child, a daughter, had grown up to womanhood without developing the symptoms of the family disease. But when she too was a wife and a mother the hereditary weakness had come out, and throughout the story she is a maniac—vulgar, malicious, and revengeful, with homicidal tendencies as well. Owing to her husband's devotion she has never been placed in an asylum, which is obviously the best place for her, and she rewards him by attempting her daughter's life and exposing his when he is suffering from pneumonia by opening the windows of his room, a deed which results in his death. She has no redeeming features, no moments of sanity, none of tenderness. All through the story she is a coarse and dangerous lunatic, and when she drowns herself no one pretends to regret her. In her daughter, Grace Hatton, the interest of the tale is supposed to centre. Grace has escaped the family curse. Bright and pretty, she has admirers in her own neighbourhood, one a man of more than double her age, Sam Myers; and the other a vulgar, cruel-minded oaf, Rivers Pearson, who, when he finds that she does not care for him, tries in one way and another to cause her unhappiness. Her first love is one Charlie Offord, a man immensely proud of his family, whom she meets when visiting Nice with some friends. When informed, by the obliging Pearson aforesaid, that there is madness in her family, he wisely gives her up, though his love for her is such that he remains unmarried for her sake, as he rather magniloquently puts it in a dying letter to her: "True is my heart still to the idol I worshipped, so true that now my name, which for centuries has lived, dies with me." Grace finds consolation in the love of Sam Myers, who, as a life-long friend of her father, knew the weakness in her mother's family, but apparently is indifferent to it. He marries her, and for a year they are happy, but the story ends with the discovery by Grace, aided by a communication from Pearson, the unforgiving, that her son will be a helpless idiot to the end of his days. The only consolation which the author offers is that the heroine's character is strengthened by suffering. As we have indicated, the tale is depressing. Nor has it literary quality to redeem it, and raise the gloomy narrative to the realm of true tragedy. Yet its very crudity gives it force. The writer seems to speak of what she has seen, not of what she has imagined. And as young people read stories who read no other thing, we may hope that the tale will be effective in preventing some love-sick man or maid from contracting a marriage which might have the effect of inflicting endless misery upon the unborn.

MOCK NURSES OF THE LATEST FASHION A.D. 1900. By FREDERICK JAMES GANT, F.R.C.S. (London: Baillière, Tindall, and Cox. 1900. Price 3s. net.)

THIS is to our minds a very unpleasant book, and it deals with a distinctly unpleasant subject, namely, the irregularities and immoralities practised by certain women who, under the assumed garb and guise of nurses, do many evil deeds. Apparently, the intention of the book is to enforce the necessity of some form of registration of nurses, but although it may be admitted that a very complete system of registration, with ample machinery for the supervision of all registered nurses during the whole of their career, ought to be effectual in preventing some of the more flagrant evils spoken of, it by no means follows that even the most complete system of espionage which nurses would be likely to tolerate would prevent every lapse from rectitude or make mere registration a certificate to character. We doubt whether any such system will ever remove the duty which lies upon every "employer" of ascertaining as far as possible the character of those whom she employs. In matters of urgency the public will always have to trust much to the reputation and standing of the "institute" or "nursing home" from which the nurse is sent, but in all other cases a knowledge of what a nurse has done, of where she has trained, and of what appointments she has held, carrying with it, as it naturally does, the power of making inquiry from any of these sources, gives the best security as to a nurse's character. When nurses come to recognise all round, what many of them have already done, that their interests are best served by the publication of an official directory, giving the details of their training and professional life, and leaving it open to the public to make what inquiries they like concerning their career, these "mock nurses" will find it a far more difficult matter than it is at present to intrude themselves upon the public.

### Everybody's Opinion.

[Correspondence on all subjects is invited, but we cannot in any way be responsible for the opinions expressed by our correspondents. No communication can be entertained if the name and address of the correspondent is not given, as a guarantee of good faith but not necessarily for publication, or unless one side of the paper only is written on.]

### TYPHOID TWICE.

"Nurse Helen" writes: I notice a letter from "A Grateful Nurse and Mother," in which she mentions her son having had enteric fever on more than one occasion. I should very much like to know if there are other cases on record of patients who have had typhoid twice, as I have been told that a person cannot have it more than once.

### PYJAMAS *versus* NIGHTSHIRTS.

"Y. S." writes: I read in a daily paper not long since that a parcel of over 700 pyjamas for the use of the wounded soldiers in South Africa had been received with great satisfaction. I could not help wondering if any trained nurse had felt gratitude on receiving these garments. My opinion, and I find it is the opinion of every nurse I have spoken to on the subject, is that pyjamas are most unsuitable in illness. In fact, in any serious illness it is almost impossible to make use of them. As many of our soldiers in South Africa will have injured limbs, I fail to see the use of pyjamas in their case. For enteric fever, also, they would be most unsuitable. I should be glad to hear the opinion of some of your readers.

### THE PROTECTION OF THE UNIFORM.

"E. J." writes: Having read with great interest Mr. Gant's book on the subject of "Mock Nurses of the Latest Fashions," I have been asked by many members of the R.B.N.A. for advice with regard to voting for an Act of Parliament to protect our uniform. Our soldiers had once to do the same. In the meanwhile, until measures can be taken to carry this out, could not every qualified nurse and those still training at hospitals have some distinctive badge, the same as our City of Dublin nurses wear, stating in full from what hospital or institution they come? This would at once show to the public that we are *bonâ fide* nurses. I am sorry to say our medals do not prevent women from copying our uniform. We nurses want this matter placed before the meeting on June 23rd, but we do not know to whom we should send in our names nor how to make this known to others, unless we can put a notice in The Hospital.

### USE OF THE OBSTETRIC BINDER.

"Nurse C." writes: I am sorry to find the usual sanitary sheet so condemned; it is not at all necessary to cut it in pieces. I always get my patients to use them. When done with I simply tie them up in brown paper, put them in an empty sanitary dust bin, set light to the lower end, and leave the lid half off. This burns beautifully, and nothing but a little smoke is seen in the garden. In my opinion Hartmann's sanitary goods are the best.

"Monthly Nurse" writes: I thank "M. L." and "L.O.S.," but I should like "L.O.S." to kindly notice that I spoke of being successful in all my cases, and also the three cases I referred to were primiparas who depend so much on their monthly nurse as in subsequent deliveries a little more is known of them. In the first case the patient had two previous miscarriages with great loss. Secondary hemorrhage came on when two weeks confined, and two days previous to my being sent for. She did well after. The second case I was unable to go to, but was told by the patient herself that it had been caused by her nurse leaving her binder off too soon and allowing her to stand too much. It is the third case which is the cause of my inquiry about binders. After a normal delivery, with occasional rise of temperature, the patient sat up on the twelfth day, when a bright discharge came on and pain. I came on the fifteenth day. The patient was up with rise of temperature, looking very distressed and both hands pressed over the abdomen; no binder or corsets on. I replaced the binder, and when the doctor came he was astonished to hear that it had been left off and symptoms of metritis and cellulitis set up. In my experience of six years I have kept the binder on according to the loss, longer if much, and always prefer the patient to be up some few days with her binder on, not for the sake of contraction only but for warmth and support, comfortably adjusted, so that the soft parts are gently held. There is not as much fear of the uterus relaxing, as must have been the case in these primiparas, but I wanted the opinion and experience of some older established nurse, as in these days of such excellent maternity training we like our patients to do well.

"E. A. B." writes: As a midwife and maternity nurse of twenty-two years' standing, may I strongly advise "Monthly Nurse" to insist, if possible, on her patients wearing a binder after their confinements. It is, in my opinion, most necessary, and is the greatest comfort to the patient. It supports the abdominal walls, which have been severely stretched by pregnancy, and gives a feeling of strength to the back, which has also been strained. And, more important still, in the first hours after labour it keeps up a certain pressure on the uterus, and thus aids the contraction which is so necessary. Many doctors, in addition, put a pad over the uterus for at least twenty-four hours after the confinement, and keep it in position by the binder. When possible I always have the binder worn for a month at least, a lighter one being used in the day after the patient begins her corsets again. If a patient is wise she will then for another month wear a proper abdominal belt during the day under the corsets, as support is needed lower down than the corsets come. The more children a woman has the more necessary it is that she should be well bound. In district work I always find this point a difficulty, the poor liking to discard the binder as soon as they are up. But by explaining matters clearly to them, and also by appealing a little to their vanity, I generally get them to keep the binder on for three weeks. I do not think doctors interfere much about the binder after the first ten days are over, but if a nurse is in any doubt about leaving it off she could always ask the doctor whether she should do so or not. A nurse must always remember that the doctor, not she, is responsible for the conduct of the case, and if she has to nurse under one who objects to the binder she can only do as he tells her, unless the patient has strong wishes on the subject, in which case she could tell the doctor that her patient wishes to be bound, and ask leave to do it.

### THE OPEN-AIR SYSTEM IN A PRIVATE HOUSE.

"M. O." writes: Can it be carried out? I am a hospital-trained nurse. Some months ago I developed lung trouble, and was thus brought face to face with the question above. My experience, though as yet it has been short, may be of value to others, so I have summarised it for the benefit of readers of the "Nursing Mirror." I am living in Guernsey. I have a sitting-room almost to myself, that is to say, anyone who does not care for so much air need not sit with me. The window is always open, generally as far as it will go. If cold I have a fire and put on a jacket or shawl, but seldom need the latter. When fine and warm enough I sit out of doors as long as possible, sufficiently wrapped up. Having a bedroom to myself the window is never moved from being right down from the top to the centre, except when the rain blows that way, when it is raised about a quarter of a yard. The drawing-down blinds are made of a material which may not be uncommon, but which I have never seen before, the pattern being open holes, allowing the air to ventilate freely through. This I find most convenient, as one does not want to feel the air when in bed, nor have too much light in the summer when the sun rises. Yet the air comes in quite as freely as if there were no blind. The foot of the bed is toward the window, and there is plenty of warm light clothing. I have never yet felt the air blowing on me, the face being the only part that feels at all what the atmosphere is like. I believe in never feeling the cold on one's body. The only meal I take with the whole family is middle-day dinner, when the room is not always as ventilated as one would like, but it does not have to be endured for long. Since undergoing this treatment, my face always flushes after having been in an unventilated room for many minutes, which I think is a proof that the want of pure air is felt at once by those whose lungs are weak.

### For Reading to the Sick.

HIM that overcometh I will make a pillar in the Temple of my God.—*Rev.* iii. 12.

**Verses.**

Now,—the sowing and the weeping,
Working hard and waiting long;
Afterward,—the golden reaping,
Harvest home and grateful song.

Now,—the long and toilsome duty,
Stone by stone to carve and bring;
Afterward,—the perfect beauty,
Of the palace of the King.

Now,—the tuning and the tension,
Wailing minors, discord strong;
Afterward,—the grand ascension
Of the Alleluia song! —*F. R. Havergal.*

**Reading.**

Every Christian life in this world, whatever its surroundings, is like "a tender plant" growing from "a root in dry ground." But we must not therefore be tempted to say that the spiritual life is impossible. There is little here for the Christian soul to draw nourishment from; there is plenty to thwart and hinder its growth. And the righteous man, "seeing and hearing" so much that is opposed to righteousness, "vexes his righteous soul from day to day." To some there are circumstances of especial uncongeniality added. But even if we have no exceptional sorrow from circumstance, we can know nothing of true spiritual life if we know nothing of this vexing. And it is a trial that increases, and must increase, as our spirituality grows.

Nevertheless we may, as Christ, live truly heavenly, spiritual, beautiful lives, all the more beautiful by contrast with their environment; overcoming all difficulties of position and atmosphere; not necessarily soured by adversity.

But how By what means? By what means do plants flourish and become the things of beauty they are, in unfavourable circumstances?

Is it not thus? They draw their strength and sweetness more from the heaven above than from the earth beneath them? The earth they live in and on; but their chief nourishment comes from the air, the light, the warmth, the moisture of heaven.

The same means are open to us. And we must live that life as He did; by intercourse with heaven, drawing celestial vitality into us; especially by the use of the especial channels appointed to convey to us the nature of God, particularly the blessed Sacrament of Holy Communion. Thus, and thus only, shall we show outward lives in any way resembling Christ's.—*W. St. Hill Bourne.*

God never sends a sorrow
Without the healing balm,
And bids us fight no battle
But for the victor's palm.
Yet we, by earth's mist clouded,
Knew not His holy will,
Till o'er the troubled waters
His voice said, "Peace, be still!"

We will go forth and conquer,
Depending on His grace;
The lowliest station near Him
Must be an honoured place.
And after battle, victory;
And after victory, rest—
Like the beloved apostle,
Upon the Master's breast.

—From "*Hymns for the Household of Faith.*"

### A Letter From One of the Army Nurse Martyrs.

### THE LATE SISTER STUART JONES.

A LIVERPOOL correspondent sends us the following extract from a letter dated April 17th from Sister Stuart Jones, formerly matron of St. John's Hospital, Uxbridge Road, whose death from enteric fever at Bloemfontein we announced last week:—

"At last we arrived at Bloemfontein, after an exciting week spent in a railway carriage, which I shall ever remember, and were kept waiting there in a waiting-room for hours for orders—hungry, thirsty, and, oh! so tired and cramped. As nurses were badly wanted, and our tenth 'General' will of course take some time to put together, we were portioned off to different hospitals in small numbers. I was sent here in charge of this hospital (Langman's), and there are but three of us Sisters to 200 patients, all very ill, and on the first evening of our arrival, just as we were retiring to bed at half-past ten, we were sent for hurriedly to help with the wounded, which were being brought from the main line to the field hospital. We worked without ceasing all night, as batch after batch was brought in, and so on next day and since, the sick and wounded constantly arriving, and enteric is rampant. We are working in tents, and, owing to severe thunderstorms during the last few days, we simply have to wade from tent to tent, the wet and mud being above our ankles. I wish I had brought some Wellington boots with me. We sleep and have our meals (when there are any to be had) in a lovely little bungalow just beyond the 'Field,' which the occupants, who were rebels, had to leave hurriedly, and of course we have commandeered it just as it was left. There is nothing to be bought in the town, which is short of water and food. We are on hospital rations, bully beef and biscuits, and on rare occasions we may get a loaf of bread, which costs 1s. 6d.; butter we never see. It is all very exciting, and though we are roughing it with a vengeance, and nearly dead with work, I am enjoying it. Everyone is so good to us—at first Langman scorned the idea of sisters and now cannot make enough of us. Dr. Conan Doyle is attached to the hospital, and all my patients are under him. He is a most charming man, and works like a brick. You should see him helping me, scrubbing up patients and feeding them like any nurse, full of humour and nonsense all the time, cheering up everyone. He told me laughingly only this morning that he was making a study of hospital nurses, and that we three might expect to see ourselves figuring prominently in his next story."

It may be added that Sister Stuart Jones, who was a native of Liverpool, received her training at Preston Infirmary, from whence she went to Dorset and Warrington Infirmaries. She was for two years a sister at Cardiff Infirmary, and was appointed matron at St. John's Hospital for Diseases of the Skin, Uxbridge Road, in January, 1899, a post she held up to the time of her departure for South Africa, or, perhaps, to put it more correctly, until her death, for through the generosity of the Hospital Committee the position was to be kept open for her to take up again on her return.

### Novelties for Nurses.

### EAU DE COLOGNE.

THE 4711 Eau de Cologne is so well known for its excellence that a further word of praise from us is hardly necessary. The perfume is remarkably fragrant, and has nothing of the "clinging" characteristics which render many other Eau de Colognes so undesirable when the first freshness has passed off. It is to be obtained at Mühlen's depot, 62, New Bond Street. Here also are to be found a variety of other delightful perfumes and toilet requisites.

### Notes and Queries.

THE Editor is always willing to answer in this column, without any fee, all reasonable questions, as soon as possible.

But the following rules must be carefully observed :—

1. Every communication must be accompanied by the name and address of the writer.
2. The question must always bear upon nursing, directly or indirectly.

If an answer is required by letter a fee of half-a-crown must be enclosed with the note containing the inquiry.

*Charge Nurse.*

(90) Will you kindly inform me what certificates are necessary for a nurse to have any prospect of being appointed as charge nurse to a fever hospital? Is it absolutely essential to have a three years' certificate from a general hospital?—*J. F. C.*

Applicants for engagements as Charge Nurses under the Metropolitan Asylums Board are required to produce certificates showing that they have satisfactorily passed a period of three yea s' training The certificates must be either from a general hospital, with a recognised training school for nurses; or a poor law infirma·y, in which systematic instruction is given and tested by subsequent examinations by an independent authority.

*Nervous Defect.*

(91) A young man of 21, who had fits as a child, has, from some central nervous defect, an arrested development of the right side, including arm and hand, leg and foot, the heel of the latter being about six inches from the ground when the toe touches it. He has not been seen by a surgeon since he was about nine years old. Any information as to the proper authorities to consult in such a case, or if any appliances which might render locomotion more easy, would be highly appreciated. 2. Could you also tell me what Weir-Mitchell treatment consists in?—*A.S.B., Bristol.*

Consult a doctor. 2. It is a special treatment of diet and prolonged rest which has been found useful in some cases of nervous disorders.

*Nurses' Co-operation.*

(92) Can you give me any information concerning the Co-operation for Nurses? Is there a Liverpool branch, and who could I write to for rules, &c.?—*E. H.*

The Nurses' Co-operation, 8, New Cavendish Street, W., is the largest association managed by nurses for nurses. There are no branches, and the secretary will give you all necessary information about it.

*Instrument.*

(93) Would you kindly tell me if there are any books published containing the names of all instruments used in surgery that would be useful to theatre and out-patient nurses?—*Sister Muriel.*

The catalogues of the best instrument makers would probably be the most exhaustive.

*Sick Bedroom.*

(94) I am a trained nurse. Have been at a case the month. Both patient and I occupy the same bed. I am obliged to take all my meals in the sick room, while there are two sitting-rooms downstairs. I have spoken to my patient about it, but she takes no notice. The room being small, I feel my health is suffering. What would you advise me to do?—*Nurse J.*

State your requirements clearly, definitely, and civilly to your patient; then, if she refuses to accede them, throw up the case. It is false economy to sacrifice your health for the sake of one case.

*Institutions.*

(95) Can you advise me the best way to join a nurses' institution, where they keep a nurse in constant employment for maternity (certificated) and general nursing?—*Nurse.*

There are many such associations advertising in the "Mirror." We can only recommend you to apply in the usual way, and to use your own discretion in accepting work from them.

*Paris.*

(96) Would you kindly give me the address of a co-operative private nursing association in Paris?—*G. D. B.*

There is none that we know of.

*Lungs.*

(97) Could you kindly tell me whether the air of Brighton or of Newhaven is fitted for a person whose lungs are delicate, and which place is preferable as a permanent home?—*S.*

There are many kinds of "delicacy." Consult a doctor.

*Offensive Breath.*

(98) "H. M." would be glad if anyone could tell her the cause of, and a simple remedy for offensive breath. Ought she to undertake maternity nursing whilst suffering in this way? Are bad teeth and chronic indigestion likely to cause it?

Consult a doctor, and most certainly have the teeth and digestion put right.

*Nursing Abroad.*

(99) Will you kindly give me the address of the Hollond Institute, also of any other institution that employs nurses for the winter season abroad?—*L. E. G.*

The Hollond Institute, 1, Tavistock Chambers, Bloomsbury, W.C. There are occasionally advertisements in the "Mirror" for nurses to go abroad.

*Travel.*

(100) Can you tell me howand where I can obtain a post to travel as nurse or nurse-companion to South Africa?—*M.*

Through the recommendation of personal acquaintances or by advertisement.

*Wet Pack.*

(101) A medical nurse would like to know the best method of doing a wet pack?

Strip the patient, wrap him in a wet sheet, and cover with blankets well tucked in. Apply for half an hour, watching the temperature; when this falls to within two degrees of that required remove the wet sheet, wrap the patient in dry blankets, and, if possible, remove him to another bed which has been prepared and placed conveniently for the purpose beforehand.

*Wardsmaid.*

(102) Would you kindly inform me what the work of a wardsmaid is in hospital, and how to apply for situation?—*E. G.*

A wardsmaid is practically the housemaid of the ward, undertaking the scrubbing, polishing, &c. Answer advertisements which generally appear in local papers.

*Dispensing.*

(103) I am 19 years old, and wish to dispense in a hospital. Have passed preliminary examination of the Pharmaceutical Society, Ireland, and have had some experience with a qualified chemist in dispensing, &c. Kindly tell me what steps I should take.—*Enquirer.*

You can only advertise, and answer advertisements. See all the medical journals.

*Convalescent Homes.*

(104) "E. R." wishes to know if you will kindly inform her where she can procure a list of Convalescent Homes, or Homes for Incurables, in Scotland.

The fullest lists are given in "Hospitals and Charities," published by the Scientific Press.

*Midwifery.*

(105) 1. Will you please tell me what is the average pay of midwives and monthly nurses? 2. Does the institution at which a monthly nurse is trained help her at expiration of term of training beyond giving a certificate of proficiency? 3. If not, will you kindly advise me what steps to take in order to obtain a situation?—*Johnnie Jones.*

1. £2 a week. 2. No. 3. Join a good association.

*Exerciser.*

(106) "Nurse" will be glad to have the address where Whiteley's Exerciser can be purchased.

Any good shop will procure you the exerciser.

*Training for Trainers.*

(107) Would you kindly let me know whether the St. George's Infirmary, Fulham Road, is a good training school for nurses; and whether, after a three years' course, a nurse could gain a good appointment elsewhere?—*C. L.*

The infirmary is an important one, and the training is for three years.

*Lunatic Attendant.*

(108) Would you be so kind as to inform me how I could obtain a situation as a lunatic attendant in an institution in any foreign country?—*B. S.*

You can apply in the usual way to any institution that may seem suitable.

*Massage.*

(109) May I ask of you, first, if there exists in London any institution where training in massage and physical culture can be had? Secondly, are there any hospitals in London where such training might be given in exchange for services rendered by a trained nurse?—*M. E. G.*

You must be careful in selecting any institution offering teaching in massage in return for services, as there are many fraudulent concerns well advertised. It would, therefore, be advisable to communicate with the Secretary of the Society of Trained Masseuses, 12, Buckingham Street, Strand, W.C. The Hospital for the Paralysed and Epileptic, Queen's Square, Bloomsbury, W.C., gives instruction in the art.

*Training.*

(110) Would you kindly tell me if it is possible for me to get into a good hospital for training with a salary first year, and which is the best hospital?—*F. W.*

A strong, intelligent girl can always find a hospital willing to train her. See the "Nursing Profession: How and Where to Train" for lists and terms.

*Outfit.*

(111) Would you kindly give me an idea of the outfit I should require for a hospital in Egypt (Port Said)?—*E. J.*

Very much the same as for a very hot summer in England, but be prepared for occasional cold nights.

*Appliances.*

(112) "Inquirer" would like to know if you would tell her where she could get a fœtal skull and female pelvis, also what they would cost. She would also like a mannikin, such as is used in hospitals for demonstrating position and movements of child in utero.

A surgical instrument maker would probably be able to supply "Inquirer" with the things she wants, or would procure them for her.

**Standard Books of Reference.**

"The Nursing Profession: How and Where to Train." 2s. net.
"The Nurses' Dictionary of Medical Terms." 2s.
"Burdett's Series of Nursing Text-Books." 1s. each.
"A Handbook for Nurses." (Illustrated.) 5s.
"Nursing: Its Theory and Practice." New Edition. 3s. 6d.
"Helps in Sickness and to Health." Fifteenth Thousand. 5s.

All these are published by THE SCIENTIFIC PRESS, Ltd., and may obtained through any bookseller or direct from the publishers, 28 & 29, Southampton Street, London, W.C.

### Travel Notes.

### LI.—THE LOIRE COUNTRY CONCLUDED.

### VOUVRAY AND ROCHECORBON.

A VERY interesting short excursion is to Vouvray, about six miles from Tours. Guide-books hardly mention this very remarkable village, almost entirely composed of cave dwellings. Hundreds of houses, if such they can be called, have been excavated in the soft yellow limestone rocks which rise to great heights on the left side of the road. All have outside staircases, and naturally all windows look in one direction. Every size and kind of dwelling is to be seen, from a peasant's hovel to a nobleman's villa. One and all are picturesque in their own particular style, and the general effect is very singular. We have something resembling it in England at Bridgnorth, but in a very modified degree and much more prosaic style. Two and a half miles further you will come upon Rochecorbon, where, amidst rich vineyards, is a tower used in more troublous times as a signal station. All about this side of Tours you will enter upon delightful haunts, walks, and short cycle runs, to fill up time after dinner.

### CHENONÇEAUX.

If time permits I should advise your staying a couple of nights at Chenonçeaux to enjoy the surrounding country, and to visit Amboise. From Tours it is only a run of twenty miles, or you can go by train if the exigencies of luggage seem to point that way. The little village presents nothing of striking interest, but the château is unique. Unfortunately it is only shown to the public on Thursdays and Sundays, so you must be careful as to your arrangements. It is a marvellous structure; the more important part is built across the river Cher, and the kitchens are placed in the massive piers of the bridge on which it rests. Begun in the time of Francis I., it teems with memories of the Valois. Here Henry II. and Diana of Poitiers gazed into each other's eyes; here Charles IX. and Henry III. spent much of that ill-tutored youth so little adapted to form the character for important destinies. Here Mary of Scotland passed some of her few happy days as a young bride, and here she has left a characteristic souvenir of her presence in a small and delicate mirror, on whose surface that fatal beauty and curiously-haunting expression of anticipated evil, so common to the Stuart type, must often have been reflected. After the death of her Royal lover Henry II., Chenonçeaux was wrested from Diana by the incensed Catherine, and bestowed upon Louise de Lorraine, the sad widow of Henry III., and eventually passed into the hands of the Vendôme family. It escaped the iconoclastic fury of the Revolutionists of 1793 because of the respect felt for its then owner, Madame Dupin, so that one has the advantage of seeing almost in perfection what a splendid house of the sixteenth century was like. Montrichard and Selles-sur-Cher must both be visited from Chenonçeaux; the later has also a fine château in very good preservation.

### AMBOISE.

A good cross road of some eight miles will take you to Amboise, which, to my mind, is more interesting even than Chenonçeaux. The position is most imposing; the massive building rises commandingly above the river and dominates the town lying at its feet. Two huge towers rising from the Rue de Montrichard were originally the only means of entrance to the castle. One of them contains an inclined spiral slope, so wide and gradual that a carriage and pair can be driven up. Between these towers runs a stone gallery, and on this in 1560 Catherine de Medicis, with her three sons, and Mary, the youthful bride of Francis, witnessed the hideous massacre of Huguenots in the town below—the forerunner of the more extensive murders of the Eve of St. Bartholomew. Mary, overcome with horror and anguish, knelt at her mother-in-law's feet to beg for mercy on the victims, and was removed in a dead faint by her husband. Further along the garden terrace is a small doorway surmounted by the royal porcupine. Here Charles VIII. met his death by striking his head against the lintel as he was going through to a game of tennis. The exquisite little Chapel of St. Hubert was built by this King, and is a marvel of delicate ornamentation. There is but little in the château itself to repay attention. The exterior is the point of interest. On the same day visit the small chateau of Clos-Lucé, where Leonardo da Vinci died during his service with Francis I.

### CHAUMONT.

Your next point will be Blois; but you must visit Chaumont *en route*. It would be a good plan to send on your luggage by train and cycle six miles to Montrichard, and then cut across north another ten to Chaumont. Spend the afternoon there, and go on ten more miles to Blois in the evening, or, if you prefer it, take train thence from Onzain near to Chaumont. The Castle of Chaumont is superbly placed overlooking the Loire. The entrance is by a quadrangle, from which a spiral stair opens. It is but little altered from the days when the wily Catherine compelled Diana of Poitiers to exchange with her and receive it in place of Chenonçeaux. It had been for some time previously Catherine's country house, and seems to breathe still her presence; the hearse-like bed remains in which the royal sleeper tossed in unresting slumber or laid awake weaving dark and sinister plots against all who opposed her will, wholesome sleep banished by the domination of her own fierce passions and still fiercer ambitions.

### ROYAL BLOIS.

Two days will suffice for visiting Blois and the neighbouring Chambord if time presses. The first morning you will devote to the castle itself, an affair of quite three hours. Every corner is full of beauty and interest, and transports one back to the stormy period of the wars of the "League." Perhaps one is most enthralled by that part where the Guise tragedy took place. It was in the bedroom of Henry III. that Henry of Guise met his death; the small alcove at the foot of the bed, where he fell stabbed by the assassins, remains unchanged. On the following day his brother, the Cardinal de Lorraine, was also murdered in a round tower on the other side of the Castle. Observe the beautiful spiral staircase of the time of Francis I. in one of the courts, his salamander profusely decorating every corner, the exquisite arcaded cloisters, and the matchless ceilings, panelled and embossed, which adorn nearly all the rooms. Chambord, though a magnificent structure, is less interesting, except in the matter of its unique central staircase, the mystery and wonder of which is beyond description. And now we must take leave of the beautiful Loire, though there is much, very much, which unfortunately we have not seen.

### TRAVEL NOTES AND QUERIES.

RULES IN REGARD TO CORRESPONDENCE FOR THIS SECTION.—All questioners must use a pseudonym for publication, but the communication must also bear the writer's own name and address as well, which will be regarded as confidential. All such communications to be addressed "Travel Editor, 'Nursing Mirror,' 28, Southampton Street, Strand." No charge will be made for inserting and answering questions in the inquiry column, and all will be answered in rotation as space permits. If an answer by letter is required, a stamped and addressed envelope must be enclosed, together with 2s. 6d., which fee will be devoted to the objects of the "Hospital Convalescent Fund." Any inquiries reaching the office after Monday cannot be answered in "The Mirror" of the current week.

TO CENTRAL ITALY (Poppy).—If you are good travellers you can go straight through to Milan. Leave Charing Cross at ten a.m., and reach Milan at nine p.m. the next day. You cannot do better than the Hotel de Rome there. Then on to Florence. As you have been there, and only mean to break your journey, go to the Hotel Minerva Piazza, St. Maria Novella. In returning from Rome, why do you say you go to Siena or Perugia? Both are equally interesting, and totally different.

LUCERNE IN THE SUMMER (Musketeer).—It is rather warm, being somewhat enclosed by the mountains. At the same time, there is always a pleasant breeze from the lake. Personally, I do not find it too hot. It all depends on whether you feel the heat much. A very favourite spot to escape summer heat is the Gütsch. There is a very good and reasonable hotel there.

## Figures and Tables

**Fig. 14. Fig. 15. f1:**